# Book Notices

**Published:** 1884-01-20

**Authors:** 


### BOOK NOTICES.

*Practitioner's Ready Reference Book,* a handy guide in office and bedside practice, by Richard J. Dunglison, A. M., M. D., author of a new School Physiology ; editor of Dunglison's Medical Dictionary and History of Medicine ; Secretary of the American Academy of Medicine, etc , etc. Third edition, thoroughly revised and enlarged. Philadelphia : P. Blakiston, Son & Co., 1883.

This is an excellent and very useful work for the practitioner, containing information on such subjects as the following—How to write prescriptions ; Use of the Hypodermic Syringe ; How to Apply Trusses ; Tables of Differential Diagnoses ; How to Apply Bandages ; How to apply immediate relief in recent accidents or illness ; Arrest of Hemorrhage, etc., etc. These and hundreds of other practical facts the author has judiciously and carefully collected from numerous sources and combined in compact form for the ready reference and use of the busy practitioner.

*Retail Druggists' Diary and Want Book.* Sixteen pages of important tables and information, scientific and political; fifty-two pages of diary, with space for each day of the year for entering memoranda; twelve pages of want book, for entering wants and purchases; ninety-four pages priced pharmaceutical catalogue, with 874 illustrations, over 14,000 items. Eighty pages priced catalogue, 193 illustrations of non-secret medicines, toilet and domestic articles, with Buyer's Address. Frederick Stearns & Co., Detroit, Mich.

The title page above copied sufficiently shows the great value and extent of the information in this work. Exceedingly useful to the druggist and scarcely less so to the physician. Frederick Stearns & Co., by whom the book is published, are manufacturing pharmacists of great enterprise and wide reputation.

*Plea for the Cure of Rupture,* or the Pathology of the Subcutaneous Operation by Injection for the Cure of Hernia, by Joseph H. Warren, A.M., M.D , Member of the British Medical Association, Member of the American Medical Association, Formerly Surgeon and Medical Director in the U. S. Army, etc. Boston: James R. Osgood & Co. London: J. & A. Churchill, 1884.

A valuable and instructive book of 112 pages.

